# Terazosin Stimulates Pgk1 to Remedy Gastrointestinal Disorders

**DOI:** 10.3390/ijms23010416

**Published:** 2021-12-30

**Authors:** Jingjing Liu, Wenyang Zhao, Chun Li, Tongyu Wu, Liang Han, Zhuozhou Hu, Xiangxiang Li, Jing Zhou, Xinping Chen

**Affiliations:** School of Pharmacy, Lanzhou University, Lanzhou 730000, China; liujj2019@lzu.edu.cn (J.L.); zhaowy19@lzu.edu.cn (W.Z.); chli2019@lzu.edu.cn (C.L.); wuty2019@lzu.edu.cn (T.W.); lhan20@lzu.edu.cn (L.H.); huzhzh20@lzu.edu.cn (Z.H.); lixx2016@lzu.edu.cn (X.L.); zhouj2016@lzu.edu.cn (J.Z.)

**Keywords:** terazosin, gastrointestinal disease, anti-inflammation, Pgk1, cell death

## Abstract

Gastrointestinal disease is the most common health concern that occurs due to environmental, infectious, immunological, psychological, and genetic stress. Among them, the most frequent diseases are gastric ulcer (GU) and ulcerative colitis (UC). DSS-induced UC and ethanol-stimulated GU models resemble the pathophysiology of human gastrointestinal disease. The current study was designed to explore the anti-oxidation, anti-inflammation, anti-cell death properties of terazosin, an α-adrenergic receptor antagonist, in vivo and in vitro. Our results indicate that terazosin dramatically activates Pgk1, and upregulates glycose metabolism, evidenced by the enhanced ATP production and higher LDH enzymatic activity. Also, terazosin significantly enhances p-AKT expression and inhibits NF-κB p65 activation through abrogating the phosphorylation of IKBα, as well as lowers Caspase-1 and GSDMD expression. The findings in this study demonstrate that terazosin exhibits anti-inflammatory effects by downregulating NF-κB-GSDMD signal pathway, along with enhancing glycolysis for gastrointestinal disease treatment. Meanwhile, we also find terazosin ameliorates ethanol-induced gastric mucosal damage in mice. Collectively, as a clinical drug, terazosin should be translated into therapeutics for gastrointestinal disease soon.

## 1. Introduction

Gastrointestinal diseases, the most common trouble in tropical countries, refer to diseases disturbing the gastrointestinal tract [1,2]. Common symptoms include vomiting and nausea, abdominal discomfort, diarrhea, acid reflux, unintentional weight loss, fatigue, swallowing difficulty, fever, black and bloody stool, gastrointestinal bleeding, and intestinal obstruction [3]. Gastrointestinal diseases are classified into two types as functional and structural. Some typical examples include dysphagia, gastric ulcer, peptic ulcer, gastroparesis, delayed gastric emptying, irritable bowel syndrome (IBS), and inflammatory bowel disease (IBD).

Ulcerative Colitis (UC), one of the inflammatory bowel disease (IBD), is an immunity-mediated chronic intestinal disorder and sometimes leads to life-threatening complications [4,5]. Until now, the etiology of UC has not been completely clarified. The occurrence of UC is thought to be complex and multifactorial, maybe due to environmental, infectious, immunological, psychological, and genetic factors [6]. Under unfavorable conditions these factors induce the release of pro-inflammatory mediators, such as reactive oxygen species, cytokines, and neutrophil infiltration, which are considered as markers in the pathogenesis of colitis [7,8]. The common clinical manifestations of ulcerative colitis mainly include abdominal pain, diarrhea, blood in the stool, fever, reduced appetite, and weight loss [9]. The clinical epidemiology investigation demonstrates that UC affects approximately 50% population in the United States, with an annual incidence of two to seven per 100,000 persons [9] and the overall incidence of the disease has remained constant over the past five decades [10]. The current clinical drugs, including 5-aminosalicylic acid, corticosteroids, immunosuppressant, biological agents, and probiotics, alleviate the symptoms, whilst the efficacy of these treatments are limited and compromised by side effects (nausea, vomiting, myelosuppression, infection, and carcinogenesis) [11,12], so the research and development of new agents remains an urgent requirement.

Recent studies strongly suggest that there is a close link between intestinal disease and metabolic genes or metabolites. Pyruvate, an intermediate product from carbohydrate metabolism, showed an anti-inflammatory effect in colitis [13]. Furthermore, short-chain fatty acids and amino acids also modulate inflammation at colonic level [14,15]. In addition, pyruvate kinase (PKM2, rate-limiting enzyme controlling the final step of glycolysis) combined with NADPH oxidases Duox and Nox (two ROS-producing enzymes) also hold a strong link with intestinal function and UC [16,17,18]. Accumulating evidences have confirmed that cellular metabolism is critical for renovating intestinal barrier function and IBD. For example, transketolase (TKT), a key player in the glycolysis and non-oxidative PPP (Pentose Phosphate Pathway), maintains intestinal ATP production and inhibits apoptosis-induced colitis in mice [18]. Moreover, reducing the glycolysis via 2-DG (2-deoxy-D-glycose, glucose inhibitor) treatment markedly blocked pro-inflammatory macrophages differentiation [19]. Beyond that, tiliroside, a flavonoid chemical compound, attenuates UC via HIF-1α-mediated reprogramming of glycolysis pathway [12]. Thus, focusing on the regulation of glucose metabolism in UC may provide new insights into clinical therapies.

Phosphoglycerate kinase 1 (Pgk1) is a key glycolytic enzyme in the creation of adenosine triphosphate [20]. It catalyzes the reversible phosphotransferase reaction from 1,3-bisphosphoglycerate (1,3-BPG) to MgADP, to generate 3-phosphoglycerate (3-PG) and the first ATP in the glycolysis pathway [21]. Hence, it plays a vital role in cell energy metabolism. Pgk1 deficiency has been characterized by chronic nonspherocytic hemolytic anemia, neurological dysfunctions, and myopathy [22]. It has been reported that terazosin (TZ) binds and activates Pgk1, thereby increasing ATP levels and curbing apoptosis [23]. The disrupted energy metabolism in IBD, combined with the fact that terazosin works as an activator of Pgk1, motivated us to propose that terazosin may ameliorate the pathophysiology of UC via upregulating glycolysis.

In the present study, we assessed the protective effect of terazosin targeting Pgk1 against gastrointestinal disease and evaluated the underlying mechanism in vivo and in vitro. Our results indicate for the first time that terazosin significantly activates Pgk1-mediated protective defenses against gastrointestinal disease in an AKT- or Cas1/GSDMD-related pyroptosis manner. The study may provide a new protective agent by enhancing glycolysis for gastrointestinal disease and highlight Pgk1 as an attractive candidate target to combat gastrointestinal disease.

## 2. Results

### 2.1. Effect of Terazosin on Cell Viability under H_2_O_2_- and 2-DG-Induced Stresses

**Figure 1:**
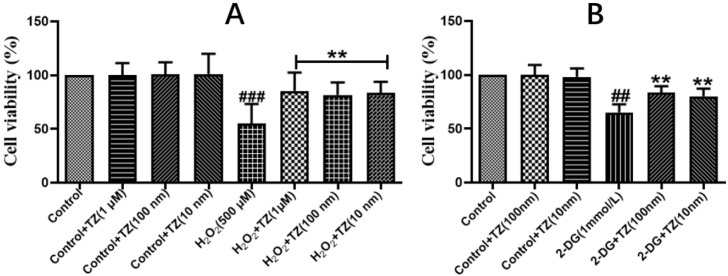
Effect of terazosin on H_2_O_2_- and 2-DG-induced Caco-2 cells. Cell viability was examined by MTT assay. (**A**) In-vitro effect of terazosin treatment on cell viability under H_2_O_2_ stress. (**B**) Effect of terazosin on cell viability under 2-DG stress. All data are represented as Mean ± SEM, ^##^ *p* < 0.01 and ^###^ *p* < 0.001 when compared to the control group, ** *p* < 0.01 when compared to H_2_O_2_ or 2-DG group.

The effect of terazosin on cell viability in Caco-2 cells was examined via MTT assay, and the results showed that treatment with terazosin (100 and 10 nM) for 24 h did not show any cytotoxicity (Figure 1A). Further results demonstrated that the cells pretreated with different concentrations of terazosin along with H_2_O_2_ (500 μmol/L) challenge exhibited better survival (Figure 1A). Hence, terazosin at 10 or 100 nM was chosen for following study. Above-mentioned results indicated that terazosin protects cells by resisting oxidative stress.

Since terazosin increases glycolysis by activating Pgk1 [23], we asked whether enhanced glycolysis also contributes to boosted stress resistance of terazosin. Herein we tried 2-DG, an allosteric inhibitor of hexokinase, to deprive glucose metabolism in cells, and found treatment with terazosin (100 and 10 nM) obviously enhanced cell viability (*p* < 0.01, Figure 1B). The findings support that terazosin protects cells by activating glycolysis.

### 2.2. Terazosin Activates Glucose Metabolism

**Figure 2:**
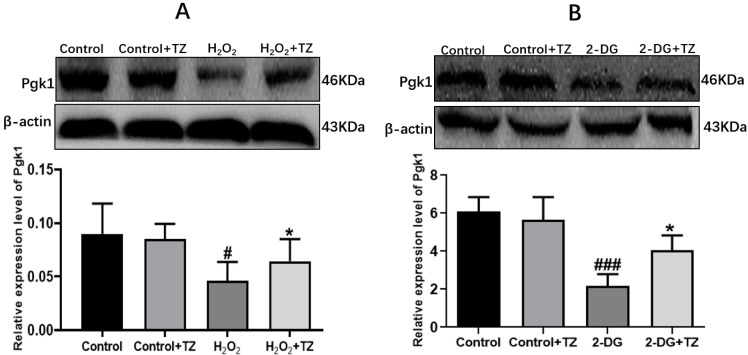
Effect of terazosin on the expression of Pgk1. (**A**) Western blot analysis of Pgk1 under H_2_O_2_ stress. (**B**) Pgk1 expression under 2-DG stress. All data are represented as Mean ± SEM, ^#^ *p* < 0.01 and ^###^ *p* < 0.001 when compared to the control group, * *p* < 0.05 when compared to the H_2_O_2_ or 2-DG group.

**Figure 3:**
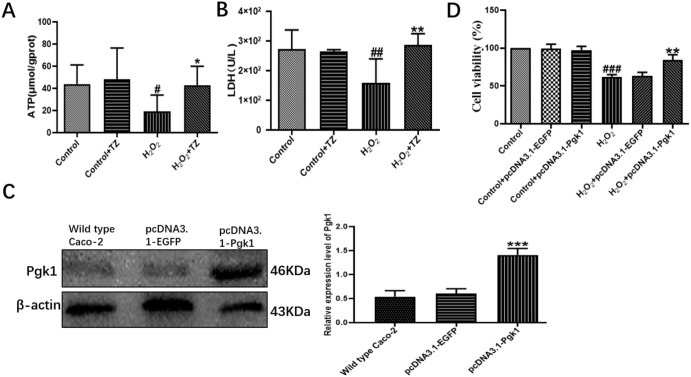
Effect of terazosin on glucose metabolism pathway. (**A**) The effect of terazosin (10 nM) on the intracellular ATP level in the cell lysate of Caco-2 cells; *n* = 8 trials. (**B**) The effect of terazosin (10 nM) on the LDH level in the cell medium of Caco-2 cells; *n* = 8 trials. (**C**) Relative expression of Pgk1 in the transfected Caco-2 cells by Western blot analysis; β-actin was used as loading control; *n* ≥ 3 trials. (**D**) Effect of Pgk1 expression on cell viability in the transfected Caco-2 cells by MTT assay. All data are represented as Mean ± SEM, ^#^ *p* < 0.05, ^##^ *p* < 0.01 and ^###^ *p* < 0.001 as compared to Control group, * *p* < 0.05, ** *p* < 0.01 and *** *p* < 0.01 as compared to H_2_O_2_ group.

Pgk1 is a key glycolytic enzyme that catalyzes the conversion of 1,3-biphosphoglycerate to 3-phosphoglycerate to generate ATP in the aerobic glycolysis pathway [24]. In order to examine if terazosin influences glucose metabolism, the content of Pgk1 was determined under the oxidative and 2-DG pressures through Western blot analysis, as well as quantification of the levels of ATP and LDH in the cells with corresponding kits. As shown in Figure 2, we found terazosin-treated cells dramatically enhance the Pgk1 expression under oxidation or 2-DG pressure (*p* < 0.01, Figure 2). Moreover, the levels of ATP (Figure 3A) and LDH (Figure 3B) in the H_2_O_2_-induced Caco-2 cells were determined to confirm whether terazosin activates glycolysis under oxidative stress. H_2_O_2_ stimulation markedly decreased ATP content (19.2475 ± 6.01 μmol/g protein, *p* < 0.05) and LDH enzyme activity (158.1395 ± 40.90 U/L, *p* < 0.01) in comparison with control, while terazosin-treatment restored ATP level (42.8696 ± 6.97 μmol/g protein) and LDH activity (286.5116 ± 17.00 U/L, *p* < 0.01) in the cells. All above-mentioned results further demonstrated that terazosin activates glucose metabolism pathway and produces energy necessary for cells survival.

### 2.3. Overexpression of Pgk1 in Caco-2 Cells

Next, cells were transfected with pcDNA3.1-Pgk1 or pcDNA3.1-EGFP, and the Pgk1 expression level was determined by Western blot. Transfection with pcDNA3.1-Pgk1 increased the expression of Pgk1 by nearly 264.15% relative to that of wild-type cells (*p* < 0.001, Figure 3C). In addition, overexpression of Pgk1 significantly enhanced cell viability under oxidative stress (*p* < 0.01, Figure 3D). The results indicated that Pgk1 overexpression directly prevented cell death due to oxidative stress.

### 2.4. Terazosin Blocks H_2_O_2_-Induced Cell Pyroptosis

**Figure 4:**
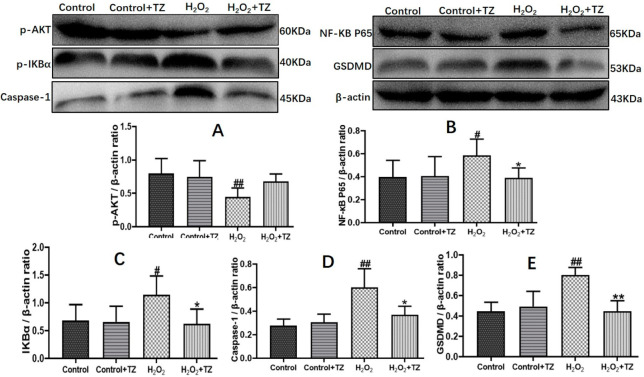
Terazosin inhibits cell apoptosis and pyroptosis in the Caco-2 cells. Terazosin influence on the levels of proteins p-AKT (**A**), NF-κB p65 (**B**), p-IKBα (**C**), caspase-1 (**D**), and GSDMD (**E**) in Caco-2 cells were verified by Western blot analysis. All data are represented as Mean ± SEM, ^#^ *p* < 0.05 and ^##^ *p* < 0.01 as compared to Control group, * *p* < 0.05 and ** *p* < 0.01 as compared to H_2_O_2_ group.

Given that UC is a chronic and idiopathic inflammatory disease, it causes immune dysregulation and release of pro-inflammatory cytokines. Previous literature showed that pyroptosis and inflammasomes are involved in various types of inflammatory diseases, including colitis [25]. Hence, we focused on whether terazosin blocks cell pyroptosis because of H_2_O_2_-stimulated injury. The results were shown in the Figure 4, H_2_O_2_ instigated a significant (*p* < 0.05) increase in the expression of p-IKBα, NF-κB p65, Caspase-1 and GSDMD, while the p-AKT expression decreased markedly in the cells compared to control. The terazosin treatment reversed the changing trend of protein levels (*p* < 0.05), supporting the conclusion that terazosin inhibits NF-κB-GSDMD axis-mediated pyroptosis.

### 2.5. Terazosin Treatment Attenuates Development of Colitis in Mice

**Figure 5:**
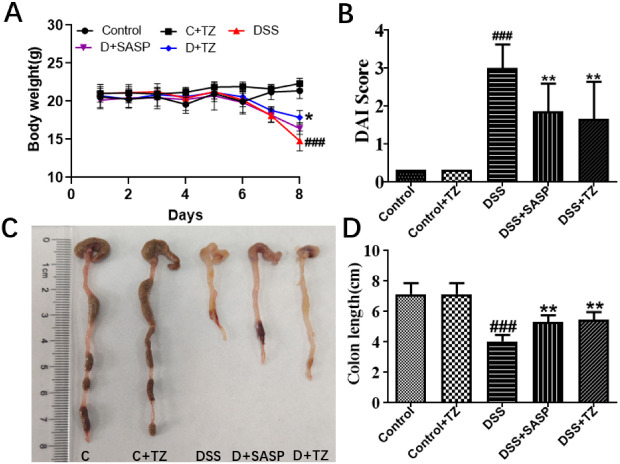
Terazosin (TZ) alleviated the clinical symptoms and representative photographs showing colon tissues in DSS-induced ulcerative colitis mice. (**A**) Body weight changes; (**B**) Disease activity index of mice treated with DSS and terazosin; (**C**,**D**) Changes of colon tissues in individual study groups and in colon length. All data are represented as Mean ± SEM, ^###^ *p* < 0.001 as compared to the control group, ** *p* < 0.01 as compared to DSS group. SASP (salicylazosulfapyridine) was taken as positive control.

To further explore the influence of terazosin on UC, the UC mouse model was built by feeding mice DSS. Clinical symptoms of UC including body weight loss, changed stool consistency, and the blood stools were determined, along with the evaluation of the disease activity index (DAI, UC disease severity). The results were shown in the Figure 5, mice administrated with 4% DSS exhibited a significant body weight loss (*p* < 0.001, Figure 5A), while the terazosin-treatment (4 mg/kg/day) group and SASP-treatment group (positive control, 80 mg/kg/day) reversed the loss on day 8 of the experiment (*p* < 0.05, Figure 5A). Moreover, we found terazosin as well as SASP markedly declined cumulative DAI scores by restoring normal stool consistency and avoiding rectal bleeding upon DSS challenge (*p* < 0.01, Figure 5B). Beyond that, the colon length is taken as a morphological parameter for the degree of inflammation of DSS-induced colitis [26]. The colon length in DSS-stimulated group was much (4.03 ± 0.18 cm, *p* < 0.001) shorter than that in the control group (7.13 ± 0.29 cm, Figure 5D). The mice with terazosin or SASP treatment displayed a notable (*p* < 0.01) increase in colon length (5.46 ± 0.23 cm and 5.33 ± 0.22 cm, respectively). Taken together, these results demonstrated that terazosin attenuates the signs of DSS-stimulated colitis in mice.

### 2.6. Terazosin Suppresses Pro-Inflammatory Cytokines in DSS-Induced Colitis in Mice

**Figure 6:**
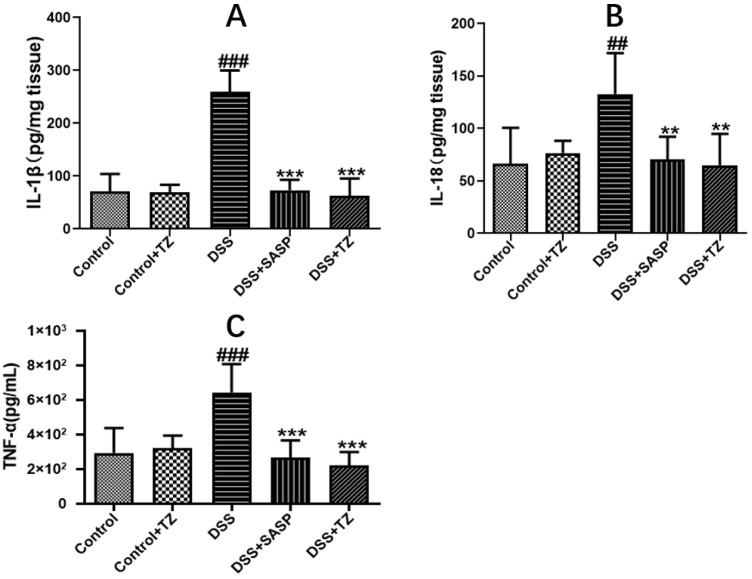
Effect of terazosin on the concentrations of pro-inflammatory cytokines. (**A**) Interleukin (IL)-1β, (**B**) Interleukin (IL)-18, and (**C**) Tumor Necrosis Factor (TNF)- α levels. All data are represented as Mean ± SEM, ^##^ *p* < 0.01 and ^###^ *p* < 0.001 as compared to the Control group, ** *p* < 0.01 and *** *p* < 0.01 as compared to DSS group. SASP (salicylazosulfapyridine) was selected as positive control.

To evaluate the impact of terazosin on pro-inflammatory response in the colon tissues of DSS-induced mice, the amounts of inflammatory cytokines were quantified. The levels of major inflammatory cytokines including IL-1β, IL-18, and TNF-α were higher in the DSS group (259.34 ± 20.42 pg/mg tissue, 132.83 ± 16.00 pg/mg tissue, and 644.93 ± 66.38 pg/mL 10% tissue homogenate, respectively) than that in the control group (70.94 ± 14.91 pg/mg tissue, 66.16 ± 14.10 pg/mg tissue, and 293.72 ± 51.03 pg/mL 10% tissue homogenate). Terazosin markedly lowered the concentrations of IL-1β (62.83 ± 14.57 pg/mg tissue, *p* < 0.001, Figure 6A), IL-18 (65.13 ± 12.11 pg/mg tissue, *p* < 0.01, Figure 6B), and TNF-α (220.11 ± 35.48 pg/mL 10% tissue homogenate, *p* < 0.001, Figure 6C) under the DSS stress. Thus, terazosin exerts an anti-colitis effects by inhibiting the release of pro-inflammatory cytokines.

### 2.7. Effects of Terazosin on SOD, MDA, Lactic Acid Amounts, and MPO Activity

As the main oxidative stress indexes, SOD and MDA play a vital role in the pathogenesis of UC. Herein, the levels of SOD and MDA in both serum and colon tissues of mice were evaluated with the related biochemical assay kits. The results were listed in the Table 1. Accordingly, DSS induced a vital reduction of SOD while increase of MDA content. For both serum and colon tissues, terazosin powerfully recovered the level of SOD with DSS treatment, further indicating a good antioxidant activity of terazosin against UC injury. In addition, we also determined the MPO activity, a reporter of neutrophil infiltration. The levels of MPO markedly increased from 58.45 ± 8.51 to 99.75 ± 11.74 U/g tissue or 56.89 ± 8.84 to 134.29 ± 13.24 U/L serum post DSS infusion. As expected, terazosin reduced the activity of MPO (34.87 ± 13.42 U/g tissue and 76.11 ± 1.02 U/L serum, respectively) by about two-fold relative to that of the DSS group. It was reported that increased lactic acid level may modulate the diarrhea of UC [27]. The lactic acid level was dramatically enhanced in the DSS group compared to that of the control, while the terazosin significantly restored the level (*p* < 0.001, Table 1). Collectively, these results suggested that terazosin ameliorates the state of oxidative stress, enhances the defense ability of intestinal mucosa, and plays a protective and therapeutic role in UC mucosa.

### 2.8. Effect of Terazosin on Gastric Ulcer in Mice

**Figure 7:**
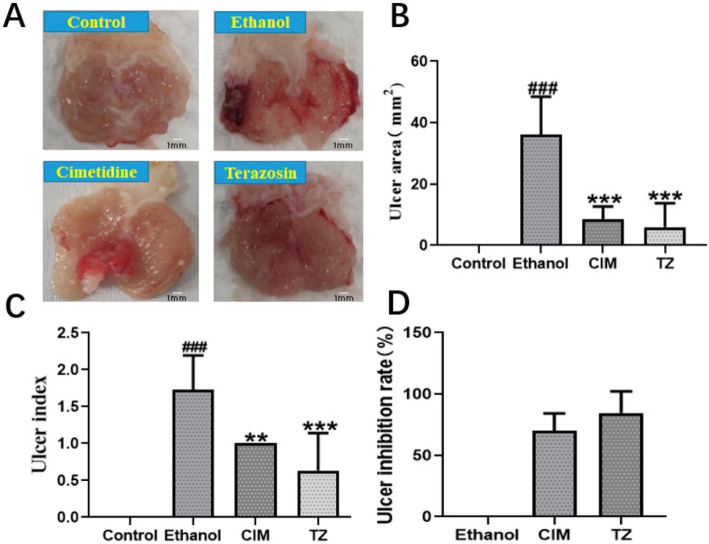
The effect of terazosin on the macroscopic appearance of the stomach mucosa (**A**), ulcer area (**B**), ulcer index (**C**), and ulcer inhibition (**D**) in ethanol-induced stomach mucosal injury in mice. The Control group exhibited no injuries to the gastric mucosa, The Ethanol group showed severe injuries to the stomach mucosa; CIM (80 mg/kg/day) and TZ (1 mg/kg/day) indicated mild disruptions of the surface epithelium in the gastric mucosa. All data are represented as Mean ± SEM, ^###^ *p* < 0.001 as compared to the Control group, ** *p* < 0.01 and *** *p* < 0.001 as compared to the Ethanol group. Cimetidine was taken as the positive control in this work.

Given that gastric ulcer and ulcerative colitis have similar pathological mechanism, and GU is also a kind of gastrointestinal disease [1], we wondered if terazosin also benefits GU in the same way. The ethanol-induced gastric ulcer model in mice was successfully built to examine the potential role of terazosin in the disease. The results were shown in Figure 7A, the ethanol alone group exerted severe gastric lesions, along with extensive visible hemorrhagic erosion resulting in high ulcer areas and high ulcer index when compared to the control. Pretreatment with 1 mg/kg/day terazosin or 80 mg/kg/day cimetidine dramatically improved gastric mucosa damage degree and decreased the ulcer areas and ulcer index compared to the control (*p* < 0.001, Figure 7B,C). Moreover, the ulcer inhibition rate of terazosin (81.57%) was higher than that of cimetidine (69.91%) (Figure 7D). This finding demonstrated that terazosin was effective in GU mice (1 mg/kg/day), and a lower dose was enough for the treatment of GU relative to the treatment of UC (4 mg/kg/day).

## 3. Discussion

This paper mainly introduced the effects of terazosin in acute gastrointestinal diseases, including Gastric ulcer (GU) and Ulcerative colitis (UC). Our results demonstrated that terazosin protects gastrointestinal disease through activating Pgk1-mediated defense system. The study may provide a new protective agent by increasing glycolysis for gastrointestinal disease and address Pgk1 as an attractive candidate target to combat gastrointestinal disease.

Gastrointestinal disease includes a group of diseases, with the most common ones being GU and UC. The primary organs affected in patients range from the esophagus, stomach, small intestine, large intestine, colon, and rectum, and the accessory organs of digestion, the liver, gallbladder, and pancreas [1]. Gastric ulcer (GU) is a localized area of erosion in the stomach lining, which resulted in abdominal pain, possible bleeding, and other gastrointestinal symptoms [28]. Excessive drinking, irregular eating, long-term mental tension, and long-term use of non-steroidal anti-inflammatory drugs (NSAIDs, such as aspirin), glucocorticoids, clopidogrel, and other exogenous factors are related to the emergence of gastric ulcer, as well as the promotion of gastric mucosal injury [29,30]. The occurrence of gastric ulcer is mostly due to the destruction of mucosal defense and repairing functions [31]. Ulcerative colitis (UC) acts as a kind of chronic inflammatory bowel diseases, the major clinical manifestations include diarrhea, abdominal pain of discomfort, and stool blood [9]. Currently, conventional medicines of UC are mainly anti-inflammatory drugs, anti-TNF-α antibody, infliximab, adalimumab, certolizumab, and natalizumabare therapy, while the treatment of UC exhibits strong side effects past a long-term use of these agents [32]. In a word, the major problem of the current therapeutic drugs against GU and UC is the limited efficacy, along with unfavorable adverse events [33,34]. Hence, an inexpensive, effective, and safe novel cell death inhibitor is on demand to bridge the gap.

Among various chemically induced gastrointestinal disease models, dextran sulfate sodium (DSS)-induced ulcerative colitis [35,36] and ethanol-stimulated gastric ulcer [37,38] in mice are widely used as pre-clinical gastrointestinal disease models since they exert similar manifestations to that of human gastrointestinal diseases. In our work, terazosin not only reversed clinical symptoms of ulcerative colitis, including weight loss, DAI score soaring, and colon length shortening (Figure 5), but also significantly ameliorated ulcer area and index, as well as increased the ulcer inhibition rate of the gastric ulcer model (Figure 7). Inflammatory response plays a vital role in the pathological process of UC and GU. Oat β-glucan was shown to prevent DSS-induced colitis by downregulating the levels of TNF-α, IL-1β, IL-6, and iNOS [39]. Meanwhile, chrysin activated peroxisome proliferator activated receptor-γ (PPAR-γ) and lowered the expression of pro-inflammatory marker genes, including TNF-α, IL-6, and CCL3, to fight indomethacin-induced gastric ulcer [40]. In the present study, we clarified that terazosin improved the health of mice and blocked inflammation in the colon tissues of DSS-induced UC (Figure 6) and gastric tissue of ethanol-stimulated GU in mice (Figure S1). Since MPO works as an index of neutrophil infiltration [41], terazosin treatment minimized infiltration of lymphocytes in the DSS-induced UC model and ethanol-stimulated GU model (Table 1 and Table S1). All results discussed demonstrated that terazosin successfully ameliorated gastrointestinal disease inflammation in mice.

Oxidative stress plays an important role in the UC and GU [42,43]. Reactive oxygen species (ROS) and reactive nitrogen species (RNS) are kept balance under normal conditions. In the presence of hydrogen peroxide (H_2_O_2_), an excessive generation of the reactive species would trigger an inappropriate mucosal immune response [44]. Previous studies showed that various oxidation related factors take part in the pathogenesis of gastrointestinal disease. MDA is the most important products of membrane lipid peroxidation, and its level can be taken as an indirect index of cumulative lipid peroxidation [45]. It is also reported that Kangfuxin treatment decreased plasma and gastric MDA levels, while increased SOD level in ethanol-induced GU mice [43]. Our results indicated that terazosin downregulates MDA level and upregulates the level of SOD, exhibiting protective effect against oxidative stress in UC and GU mice (Table 1 and Table S1).

Apart from ROS and oxidative stress, cellular ATP level is also an important determinant for intestinal epithelial apoptosis [18]. Glucose is a major source of ATP and can be metabolized by both glycolysis and PPP pathway. Among them, glycolysis converts glucose to pyruvate and lactic acid with a series of enzymes in the cytoplasm [12]. Accordingly, here in our study, terazosin treatment enhanced the levels of ATP and LDH in the H_2_O_2_-induced Caco-2 cells (Figure 3A,B) and ethanol-stimulated GES-1 cells (Figure S2A,B). Simultaneously, the terazosin-treated group could decrease the lactic acid levels in the colon tissue (Table 1) and gastric tissue (Table S1), further indicating that terazosin protects gastrointestinal disease through activating glycolysis. Pgk1 acts as a key glycolytic enzyme in the creation of adenosine triphosphate [20]. It catalyzes the reversible phosphotransferase reaction from 1,3-bisphosphoglycerate (1,3-BPG) to MgADP, to generate 3-phosphoglycerate (3-PG) and the first ATP in the glycolysis pathway [21], by doing so it plays a vital role in cell energy metabolism, that motivated us to suppose Pgk1 centers in the pathogenesis of the gastrointestinal disease. To investigate whether targeting Pgk1 display any effect on gastrointestinal diseases, we hypothesized that gain-function of Pgk1 influences the pressure resistance of intestinal epithelial cells. In the work, we transfected Caco-2 cells with the plasmid pcDNA3.1-Pgk1, with the pcDNA3.1-EGFP as the control. Upon H_2_O_2_ stimulation, cells overexpressing Pgk1 exhibited dramatic reduction of cell death (Figure 3D). The data above demonstrate that terazosin targets Pgk1 to activate glycolysis for treating gastrointestinal diseases.

**Figure 8:**
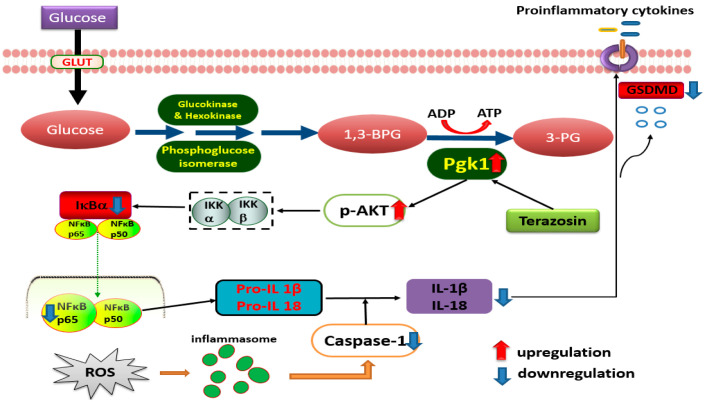
Schematic presentation illustrating the possible pathways of UC induction and targets of ulcer protection by terazosin. Terazosin activates Pgk1, followed by stimulation of Akt signaling, finally downregulates Caspase-1 to block pyroptosis.

In order to further explore the detailed mechanism of terazosin regulation on NF-κB-GSDMD axis-mediated pyroptosis, p-AKT, p-IKBα, NF-κB p65, Caspase-1, and GSDMD were detected by Western blotting. Pyroptosis, a highly inflammatory form of lytic programmed cell death, with initiation via activation of caspase family, including caspase-1, caspase-4, caspase-5, and caspase-11, can be triggered by various diseases, such as ulcerative colitis [46]. As a critical nuclear transcription factor, NF-κB not only regulates pro-inflammatory genes (TNF- α, IL-1β, and IL-6) [47], but also controls the transcription of gasdermin D (GSDMD), which was identified by 2 independent screening approaches as a key effector of pyroptosis [48,49,50]. In most cells, NF-κB is present as a latent, inactive, and IκB-bound complex in the cytoplasm, and phorylation of IκB (IκBα and p100) is essential for NF-κB activation [51]. IκBβ is also targeted for phosphorylation on Ser19 and Ser23 through binding to the IKK complex (the core element of NF-κB cascade) [51]. The activity of NF-κB is primarily regulated by interaction with inhibitory IKB proteins, and its inactivation can alleviate the severity of UC [26,52]. Here, we observed that terazosin treatment significantly increased the phosphorylation of IκBα and subsequent translocation NF-κB p65 into the nucleus (*p* < 0.05, Figure 4). Meanwhile, we also found terazosin obviously blocked the release of IL-1β and IL-18, as well as attenuated pyroptosis in DSS-induced UC mice (*p* < 0.001 and *p* < 0.01, Figure 6). Reports indicated that the production of IL-1β and IL-18 depends on the Caspase-1 activity, our results confirmed terazosin treatment markedly inhibited Caspase-1 and GSDMD expression in H_2_O_2_-induced Caco-2 cells (*p* < 0.05, Figure 4). We also examined that the enhancement of p-AKT could reduce the expression of pyroptosis-related protein and alleviate UC injury, just consistent with previous report [53]. The findings show that terazosin protects UC by suppressing NF-κB-GSDMD axis-mediated pyroptosis through activating Pgk1, the new signaling pathway was illustrated in Figure 8.

Aside from terazosin, we discovered other α-adrenergic blockers including alfuzosine and prazosin exert similar potency against ulcerative colitis and gastric ulcer in mice. The results were shown in the Figures S3 and S4, alfuzosine (2 mg/kg/day) and prazosin (5 mg/kg/day) ameliorated clinical symptoms of ulcerative colitis, including Disease Activity Index score and colon length shortening, but also dramatically attenuated ulcer area and index, as well as increased ulcer inhibition rate (77.35 and 77.37%, respectively) of gastric ulcer model. Meantime, we performed an in vitro assay and found prazosin and alfuzosine strongly enhanced Pgk1 activity in other work. Combining with the empirical results, we supposed alfuzosine, prazosin and terazosin target Pgk1 to enhance glycolysis to block cell death in ulcerative colitis and gastric ulcer.

In summary, this research for the first time demonstrates clearly that terazosin exerts anti-oxidative, anti-inflammatory and anti-cell death effects on gastrointestinal disease in vivo and in vitro through blocking the pro-inflammatory cytokines, which are involved in the NF-κB-GSDMD axis-mediated pyroptosis pathways. Also, we find terazosin targets Pgk1 to benefit gastrointestinal disease by activating glycolysis, that may revolutionize the future therapy of the gastrointestinal diseases. Terazosin is a medication used in the management and treatment of benign prostatic hyperplasia and essential hypertension, an approved drug in the clinic with well-established pharmacokinetic and safety profiles in humans. These findings here would accelerate its potential development and repurposing as a new clinical drug. Combination with other clinical drugs would produce synergetic effect by targeting multiple targets.

## 4. Materials and Methods

### 4.1. Reagents and Chemicals

Terazosin (TZ, purity ≥ 98%) and Dextran Sulfate sodium (DSS, MW~40kDa) were purchased from Aladdin Biotechnology Co., Ltd.(Shanghai, China); 3-(4,5-Dimethyl-2-thiazolyl)-2,5-diphenyl-2-H-tetrazolium bromide (MTT) and dimethylsulfoxide (DMSO) were from Solarbio Life Science (Beijing, China); Primary rabbit antibodies against Pgk1, caspase-1, GSDMD, p-AKT (T450), and β-actin were purchased from BOSTER Biological Technology Co., Ltd. (Beijing, China); Antibodies against NF-κB p65 (3033) and p-IKBα (5209) were purchased from Cell Signaling Technology (Beverly, MA, USA).

### 4.2. Cell Lines and Cell Culture

Human colorectal adenocarcinoma Caco-2 cell was obtained from Procell Life Science & Technology (Wuhan, China), that has been widely used as an in vitro model of the intestinal epithelium [54]. Caco-2 cells were cultured in RPMI 1640 medium supplemented with 10% fetal bovine serum and 1% antibiotic-antimycotic solution (Tips Biological Co., Ltd. Shanghai, China), which were maintained at 37 °C in a humidified 5% CO_2_ atmosphere (Biobase Co., Ltd, Jinan, China).

### 4.3. Cytotoxicity and Viability Assay

Cell viability was performed according to the MTT reduction assay method. Caco-2 cells were seeded in a 96-well plate at a density of 1 × 10^4^ cells per well. Twelve hours (h) post-seeding, cells were pretreated with 100 and 10 nmol/L terazosin (prepared in medium and filter-sterilized with a 0.22-μm filter), and then the complete medium containing H_2_O_2_ (500 μM) or 2-DG (glucose metabolism inhibitor, 100 μM) was replaced for 24 h. Then, 50 μL of MTT solution (2 mg/mL, dissolved in PBS buffer) was added to each well and incubated for 4 h at 37 °C in a humidified incubator with 5% CO_2_ atmosphere. Finally, the media in the cells were removed and 100 μL of DMSO was added to dissolve formazan, along with incubation for 10 min at room temperature. The absorbance of each well at 405 nm was examined by a Microplate Reader (DeTie Experimental Equipment Co., Ltd. Nanjing, China).

### 4.4. Quantification of Released LDH and Intracellular ATP in Caco-2 Cells

Caco-2 cells were seeded at a destiny of 1 × 10^5^ cells per well into a 24-well plate, followed by treatment with 10 nmol/L terazosin for 24 h. Then the cells were challenged with 500 μmol/L H_2_O_2_ for 24 h. After incubation, the medium was collected and applied for LDH assay (Nanjing Jiancheng, Nanjing, China). Finally, the ATP concentrations in cells were measured at 636 nm by Ultraviolet spectrophotometer.

### 4.5. Transfection of Plasmids

Pgk1 expression vector, pcDNA3.1-Pgk1 was constructed by cloning full-length wild-type human *Pgk1* coding sequence into pcDNA3.1. The other plasmid pcDNA 3.1-EGFP was taken as a control in this work. Transient transfection with Lipofectamine^TM^ 8000 reagent (Beyotime, Shanghai, China) was carried out according to the manufacturer’s instructions. Briefly, 5 × 10^5^ cells per well were seeded in a 6-well plate one day prior to transfection. The following day cells reached 70–80% confluence. To each well, a mixture of 2.5 μg of DNA, 125 μL of medium without FBS, and 4 μL of Lipofectamine^TM^ 8000 was added and incubated at 37 °C and 5% CO_2_. Finally, the stably transfected Caco-2 cells clones were selected with 0.2 mg/mL G418 antibiotics among 3 weeks.

### 4.6. Western Blot

Caco-2 cells were seeded in 10-cm^2^ culture dish at a density of 1 × 10^6^ cells per well and then treated with terazosin, as well as H_2_O_2_ for indicated times. After that, the cells were washed with ice-cold PBS three times and extracted for total protein using RIPA buffer containing 1% PMSF. An equal amount of protein (20 μg) was resolved by 10% sodium dodecyl sulfate-polyacrylamide gel electrophoresis (SDS-PAGE) and transferred onto a 0.22-μm polyvinylidene fluoride (PVDF) membrane. And then the membrane was blocked with 5% skimmed milk for 2 h at the room temperature and incubated with primary antibodies overnight at 4 °C, including anti-Pgk1 (1:1000 dilution in 5% non-fat milk); anti-p-AKT (1:1000); anti-NF-κB p65 (1:500); anti-p-IKBα (1:400); anti-caspase-1 (1:500) and anti-GSDMD (1:500). Finally, the secondary antibody marked by horseradish peroxidase (1:2000) was incubated for 1 h at room temperature and exposed to enhanced chemiluminescence reagents. β-actin was taken as a loading control in the experiments. The signals were captured and the intensity of the bands was quantified by ImageJ.

### 4.7. Experimental Animals

Male C57BL/6N mice weighing 18~22 g were obtained from Lanzhou veterinary research institute, Chinese academy of agriculture science (SCXK-2020-0002, Gansu, China). A total of 50 mice were housed under controlled conditions (25 ± 1 °C, 45–50% humidity, and 12 h light/dark) and allowed ad libitum and water, as well as adaptation to the environment for 5 days before the experiments. All animal experiments were approved by Animal Ethics Committee of Lanzhou University (SYXK (Gan) 2018-0002 and the date of approval is 10 December 2019).

### 4.8. Experimental Design 

Experimental ulcerative colitis in mice was established by feeding the animals with 4% DSS in drinking water for 7 consecutive days [41]. All mice were randomly divided into five groups (10 animals for each group) as follows: control group (animals were fed with normal drinking water); control + TZ (animals were administered terazosin at a dose 4 mg/kg/day through intraperitoneal injection and fed normal drinking water from day 1 to day 7); DSS group (animals were fed 4% DSS in drinking water from day 1 to day 7) as disease model; DSS + SASP group (80 mg/kg/day of salazosulfapyridine by oral gavage with 4% DSS in drinking water from day 1 to day 7) as positive control; DSS + TZ group (4 mg/kg/day terazosin was administrated through intraperitoneal injection with 4% DSS in drinking water from day 1 to day 7). On the 8th day, the colon tissues from all animals were isolated for length measurement. The serum and the colon tissues were collected and stored at −80 °C for further biochemical analysis.

### 4.9. Assessment of Disease Activity Index

The clinical assessment of disease index was carried out as previously described [55]. Body weight loss, stool consistency, and fecal bleeding were assessed to evaluate the disease severity and a scoring system was assigned to each disease symptom to calculate the disease activity index (DAI). The detailed steps are as follows: the DAI was assessed as the sum of the body weight loss (scored as: 0, none; 1, 1~5%; 2, 6~10%; 3, 11~15%; 4, over 15%), the stool consistency (scored as: 0, well-formed pellets; 2, loose stools; 4, diarrhea), and the fecal bleeding (scored as: 0, negative fecal occult blood test; 2, positive fecal occult blood test; 4, gross bleeding).

### 4.10. Measurement of Oxidative Stress Indexes in Serum and Colon Tissue

SOD and MDA have been considered as biological markers of oxidative stress [56], and both were evaluated with biochemical kits following the manufacturer’s instructions (Nanjing Jiancheng, Nanjing, China). Briefly, the serum was obtained from the blood plasma and the 10% tissue homogenate was provided from colon tissue post centrifugation for estimation of antioxidant enzymes.

### 4.11. Myeloperoxidase Activity Assay in Serum and Colon Tissue

Myeloperoxidase (MPO) acts as a marker of neutrophil infiltration, and its activity was determined as described in published literature [41]. Colon tissue was homogenized with ice-cold 0.9% saline containing 1% protease inhibitor cocktail to obtain 10% tissue homogenates, and that was centrifuged at 12,000 g for 20 min at 4 °C for the measurement of MPO activity with the MPO assay kit (Nanjing Jiancheng, Nanjing, China).

### 4.12. Measurement of L-LA in Serum and Colon Tissue

Lactic acid acts as a metabolite of anaerobic oxidation of glucose, and its amount reflects the state of tissue oxygen supply and metabolism.

### 4.13. Determination of Cytokines in Colon Tissue

The concentrations of TNF-α, IL-1β, and IL-18 in colon tissue homogenates were detected with mouse specific ELISA kits (Elabscience, Wuhan, China).

### 4.14. Ethanol-Induced Gastric Ulcer

Replication of alcoholic gastric ulcer model in mice: forty male KM mice, 3 months of age, were purchased from the experimental animal center of Lanzhou veterinary research institute, Chinese academy of agricultural sciences (Lanzhou, China), and adapted to the environments for 7 days before the experiment. Food and drinking water were supplied ad libitum. The animals were randomly divided into the four groups with 10 per group. Prior to the experiment, all animals were fasted for 24 h with free access to water. Group 1 (vehicle control) and group 2 (ulcer group) were administered with 0.9% saline (1 mL/100 g); group 3 (positive control) was fed with 80 mg/kg/day cimetidine orally; group 4 was orally administered with the 1 mg/kg/day terazosin. Post 2 h, group 1 mice were infused with 0.9% saline (1 mL/100 g), and group 2–4 mice were given the ethanol (1 mL/100 g) for 2 h according to the method of previous report with small modifications [57]. Finally, the serum and the stomachs were obtained and stored at −80 °C for further analysis.

To evaluate the stomach injury, the mice were sacrificed and the stomachs were taken out slowly, after rinsing with 0.9% saline, the gastric mucosal ulcer were ready for observation [58]. Firstly, the ulcer areas (mm^2^) were calculated with the transverse and longitudinal diameters of the ulcer, as shown in formula 1. Then, the ulcer inhibition rate (%) was counted by formula 2. Accordingly, the mean value of the sum of ulcer points in each group was taken as the ulcer index (0 for healing, 1 for superficial mucosal erosion, 2 for deep ulcer or transmural necrosis, and 3 for perforation or penetrating ulcer).

Ulcer area (mm^2^) = maximum length diameter of ulcer × maximum width diameter perpendicular to the maximum length diameter (1)

Inhibition%= [(UA control-UA treated)/UA control] × 100(2)

### 4.15. Statistical Analysis

The statistical analysis was conducted with SPSS 25.0 statistical software. All of the data were presented as mean ± SEM, unless stated otherwise. Student’s *t*-test and one-way ANOVA were applied for statistical analysis involving two- or multiple-experimental-group comparisons, respectively. *P* < 0.05 was considered as statistically significance.

## Figures and Tables

**Table 1:** Effect of the oral treatment with terazosin on the lactic acid (LA), superoxide dismutase (SOD) and malondialdehyde (MDA) levels, as well as myeloperoxidase (MPO) activity of DSS-induced colitis in mice.

		Colon Tissue				Serum		
Groups	Dose	Lactic Acid (mmol/gprot)	MPO(U/g)	SOD (U/mgprot)	MDA (nmol/mgprot)	MPO(U/L)	SOD(U/mL)	MDA (nmol/mL)
Control	-	0.04 ± 0.004	58.45 ± 8.51	83.57 ± 2.38	58.45 ± 8.51	56.89 ± 8.84	83.57 ± 2.38	3.07 ± 0.68
Control + TZ	4 mg/kg/day	0.04 ± 0.004	52.72 ± 10.42	81.95 ± 3.92	52.72 ± 10.42	48.45 ± 6.57	81.95 ± 3.92	0.52 ± 0.33
DSS	-	0.13 ± 0.006 ^###^	99.75 ± 11.74 ^###^	69.44 ± 5.24 ^###^	99.75 ± 11.74 ^##^	134.29 ± 13.24 ^###^	69.44±5.24 ^#^	39.26 ± 4.47 ^###^
DSS + SASP	80 mg/kg/day	0.09 ± 0.009 ***	18.26 ± 4.45 ^***^	83.29 ± 3.52 ***	18.26 ± 4.46 ***	61.06 ± 6.37 ***	83.29 ± 3.52 *	6.86 ± 1.09 ***
DSS + TZ	4 mg/kg/day	0.08 ± 0.006 ***	34.87 ± 13.42 ***	80.67 ± 3.81 ***	34.88 ± 13.42 ***	76.11 ± 1.02 **	80.67 ± 3.81	2.76 ± 1.34 ***

The results are repressed as mean ± SEM (*n* ≥ 3). Statistical analyzes were performed using one-way analysis of variance (ANOVA) followed by an LSD-t test. *^#^ p* < 0.05, ^##^ *p* < 0.01, ^###^ *p* < 0.001 when compared with the Control group; * *p* < 0.05, ** *p* < 0.01, *** *p* < 0.001 when compared with the DSS group; SASP: salicylazosulfapyridine.

## Data Availability

The data are deposited on local resources and are available upon request.

## Supplementary Materials

The following supporting information can be downloaded at: www.mdpi.com/article/10.3390/ijms23010416/s1.
