# Supplementary material for: Terazosin Stimulates Pgk1 to Remedy Gastrointestinal Disorders
Source: Int J Mol Sci. 2021 Dec 30;23(1):416. doi: 10.3390/ijms23010416 (PMC8745693; doi:10.3390/ijms23010416)
Supplement: Supplementary file 1 [file ijms-23-00416-s001.zip › ijms-1526274-supplementary.pdf]

Supporting Materials

Additional Supporting Information may be found in the online version of this article:

Table.S1. Effect of the oral treatment with terazosin on the lactic acid (LA), superoxide dismutase (SOD) and malondialdehyde (MDA) levels, as well as myeloperoxidase (MPO) activity of ethanol-induced gastric ulcer in mice.

Figure.S1. Effect of terazosin on the concentrations of pro-inflammatory cytokines in stomach tissue of GU mice. (A) Interleukin (IL)-1β, (B) Interleukin (IL)-18 and (C) Tumor Necrosis Factor (TNF)- α levels.

Figure.S2. Effect of terazosin on glucose metabolism pathway. (A) The effect of terazosin (10 nM) on the intracellular ATP level in the cell lysate of GES-1 cells; n=8 trials. (B) The effect of terazosin (10 nM) on the LDH level in the cell medium of GES-1 cells; n=8 trials. All data are represented as Mean ± SEM, $^{\#\#}p<0.01$ and $^{\#\#\#}p<0.001$ as compared to Control group.

Figure.S3. Effect of Alfuzosine (AZ, 2 mg/kg/day, i.p) on Ethanol-induced gastric ulcer and DSS-stimulated ulcerative colitis in mice. (A) The macroscopic appearance of the stomach mucosa, (B) Ulcer index, (C) Ulcer area, (D) Disease activity index of mice treated with DSS and alfuzosine; (E&F) Changes of colon tissues in individual study groups and in colon length. All data are represented as Mean ± SEM, $^{\#\#\#}p<0.001$ as compared to Control group, $^{*}p <0.05$, $^{**}p <0.01$ and $^{***}p <0.01$ as compared to Ethanol or DSS group. Cimetidine and SASP (salicylazosulfapyridine) were taken as the positive control in this work.

Figure.S4. Effect of Prazosin (PZ, 5 mg/kg/day, Oral.) on Ethanol-induced gastric ulcer and DSS-stimulated ulcerative colitis in mice. (A) The macroscopic appearance of the stomach mucosa, (B) Ulcer index, (C) Ulcer area, (D) Disease activity index of mice treated with DSS and prazosin; (E&F) Changes of colon tissues in individual study groups and in colon length. All data are represented as Mean ± SEM, $^{\#\#\#}p<0.001$ as compared to Control group, $^{**}p <0.01$ and $^{***}p <0.01$ as compared to Ethanol or DSS group. Cimetidine and SASP (salicylazosulfapyridine) were taken as the positive control in this work.

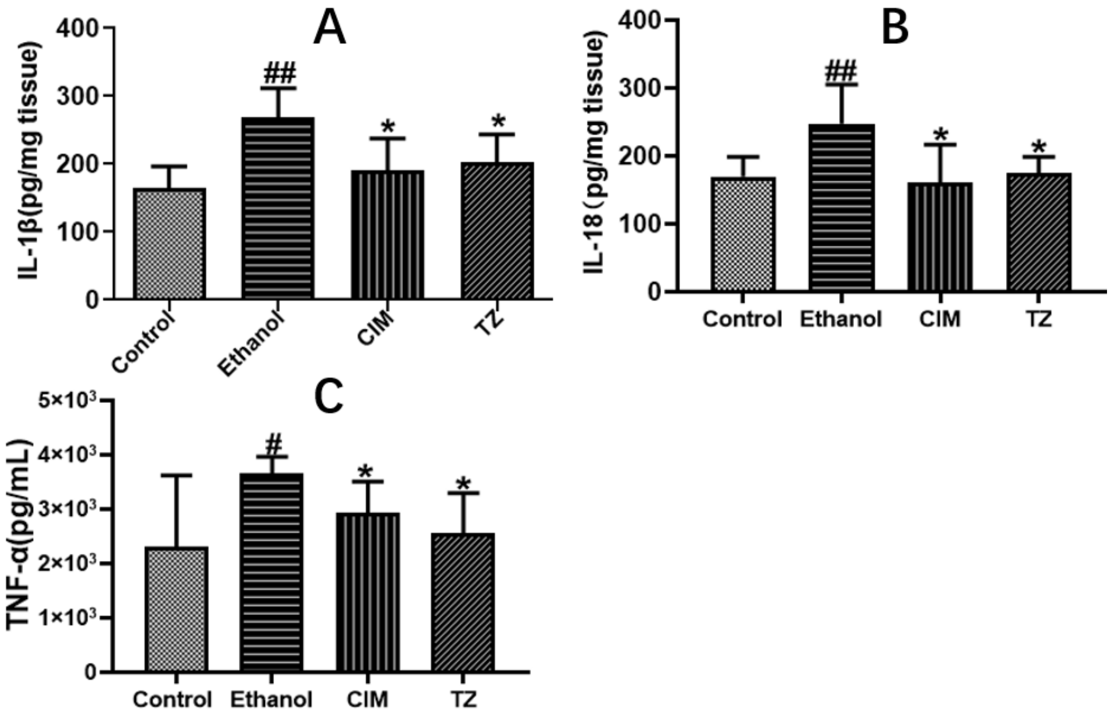

Figure.S1 Effect of terazosin on the concentrations of pro-inflammatory cytokines in stomach tissue of GU mice. (A) Interleukin (IL)-1β, (B) Interleukin (IL)-18 and (C) Tumor Necrosis Factor (TNF)- α levels. All data are represented as Mean ± SEM, $^{\#}p<0.05$ and $^{\#\#}p<0.01$ as compared to Control group, $^{*}p<0.05$ as compared to Ethanol group. CIM (cimetidine) was selected as positive control.

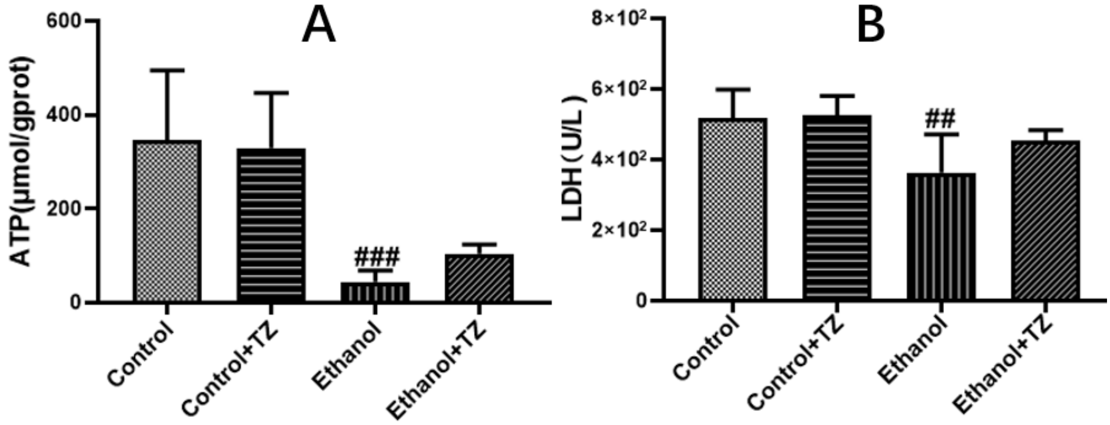

Figure.S2 Effect of terazosin on glucose metabolism pathway. (A) The effect of terazosin (10 nM) on the intracellular ATP level in the cell lysate of GES-1 cells; n=8 trials. (B) The effect of terazosin (10 nM) on the LDH level in the cell medium of GES-1 cells; n=8 trials.

All data are represented as Mean ± SEM, $^{\#\#}p<0.01$ and $^{\#\#\#}p<0.001$ as compared to Control group.

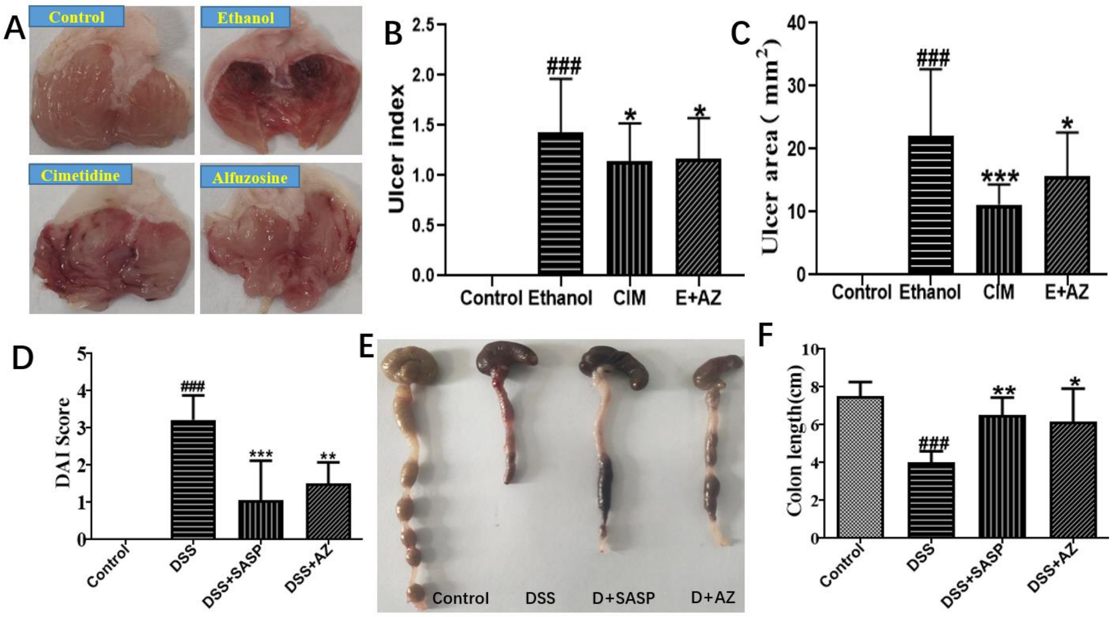

Figure.S3 Effect of Alfuzosine (AZ, 2 mg/kg/day, i.p) on Ethanol-induced gastric ulcer and DSS-stimulated ulcerative colitis in mice. (A) The macroscopic appearance of the stomach mucosa, (B) Ulcer index, (C) Ulcer area, (D) Disease activity index of mice treated with DSS and alfuzosine; (E&F) Changes of colon tissues in individual study groups and in colon length. All data are represented as Mean ± SEM, $^{\#\#\#}p<0.001$ as compared to Control group, $^{*}p<0.05$, $^{**}p<0.01$ and $^{***}p<0.01$ as compared to Ethanol or DSS group. Cimetidine and SASP (salicylazosulfapyridine) were taken as the positive control in this work.

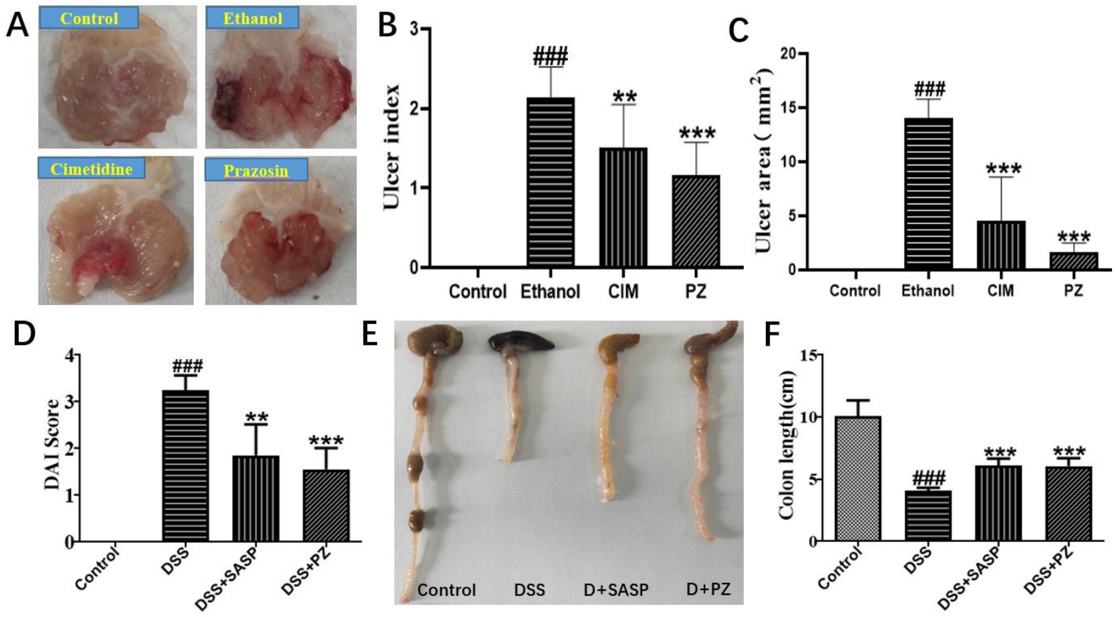

Figure.S4 Effect of Prazosin (PZ, 5 mg/kg/day, Oral.) on Ethanol-induced gastric ulcer and DSS-stimulated ulcerative colitis in mice. (A) The macroscopic appearance of the stomach mucosa, (B) Ulcer index, (C) Ulcer area, (D) Disease activity index of mice treated with DSS and prazosin; (E&F) Changes of colon tissues in individual study groups and in colon length. All data are represented as Mean ± SEM, $^{\#\#\#}p$<0.001 as compared to Control group, $^{**}p$ <0.01 and $^{***}p$ <0.01 as compared to Ethanol or DSS group. Cimetidine and SASP (salicylazosulfapyridine) were taken as the positive control in this work.

Table.S1 Effect of the oral treatment with terazosin on the lactic acid (LA), superoxide dismutase (SOD) and malondialdehyde (MDA) levels, as well as myeloperoxidase (MPO) activity of ethanol-induced gastric ulcer in mice.

		Stomach tissue				Serum		
Groups	Dose	Lactic acid (mmol/gprot)	MPO(U/g)	SOD(U/mgprot)	MDA(nmol/mgprot)	MPO(U/L)	SOD(U/mL)	MDA (nmol/mL)
Control	-	0.07±0.003	30.69±5.55	136.55±5.58	30.69±5.55	62.65±10.36	89.30±3.51	2.78±0.49
Ethanol	-	0.13±0.017$^{\#\#}$	58.81±8.77$^{\#}$	74.83±12.58$^{\#\#\#}$	58.81±8.77$^{\#}$	250.74±70.86$^{\#\#}$	49.19+±11.09$^{\#\#\#}$	5.04±0.30$^{\#\#}$
Ethanol+CIM	80mg/kg/day	0.09±0.013*	40.67±6.28	127.90±6.78***	40.68±6.28	143.06±15.12*	85.02±6.66**	1.63±0.47***
Ethanol+TZ	1mg/kg/day	0.09±0.014*	40.67±6.28	133.97±8.28***	42.27±3.55	82.01±5.14**	71.64±3.00*	3.61±0.18*

The results are repressed as mean ± SEM (n≥3). Statistical analyzes were performed using one-way analysis of variance (ANOVA) followed by LSD-t test.

$^{\#}p$ < 0.05, $^{\#\#}p$ < 0.01, $^{\#\#\#}p$ < 0.001 when compared with Control group;

$^{*}p$ < 0.05, $^{**}p$ < 0.01, $^{***}p$ < 0.001 when compared with Ethanol group;

CIM: Cimetidine.
